# Max Well-Being: a modular platform for the gamification of rehabilitation

**DOI:** 10.3389/frobt.2024.1382157

**Published:** 2024-05-31

**Authors:** Maxwell Kennard, Modar Hassan, Yukiyo Shimizu, Kenji Suzuki

**Affiliations:** ^1^ Graduate School of Science and Technology, University of Tsukuba, Tsukuba, Japan; ^2^ Institute of Systems and Information Engineering, University of Tsukuba, Tsukuba, Japan; ^3^ Department of Rehabilitation Medicine, Faculty of Medicine, University of Tsukuba, Tsukuba, Japan

**Keywords:** gamification, rehabilitation, adherence, usability, telerehabilitation

## Abstract

This study proposes a modular platform to improve the adoption of gamification in conventional physical rehabilitation programs. The effectiveness of rehabilitation is correlated to a patient’s adherence to the program. This adherence can be diminished due to factors such as motivation, feedback, and isolation. Gamification is a means of adding game-like elements to a traditionally non-game activity. This has been shown to be effective in providing a more engaging experience and improving adherence. The platform is made of three main parts; a central hardware hub, various wired and wireless sensors, and a software program with a stream-lined user interface. The software interface and hardware peripherals were all designed to be simple to use by either a medical specialist or an end-user patient without the need for technical training. A usability study was performed using a group of university students and a group of medical specialists. Using the System Usability Scale, the system received an average score of 69.25 ± 20.14 and 72.5 ± 17.16 by the students and medical specialists, respectively. We also present a framework that attempts to assist in selecting commercial games that are viable for physical rehabilitation.

## 1 Introduction

Rehabilitation refers to a group of treatments intended to improve functionality and lessen disability in people with medical issues as they interact with their surroundings. Certain medical issues may cause impairments to a person’s physical, emotional, or cognitive functioning Cieza (2019). Over the last 30 years, individuals in need of rehabilitation have increased by 63% globally, and the most common cause is due to musculoskeletal disorders Cieza et al. (2020). Therefore, this research will focus on the augmentation of conventional physical rehabilitation.

In recent years there has been a growing trend of “telehealth” and home-based rehabilitation services Shaver (2022); Velez et al. (2023). This shift may be exacerbated by the fact that limited time and resources make it difficult to effectively treat every patient in a hospital setting Fiscella and Epstein (2008). In Japan, the period of rehabilitation covered by insurance is limited Ministry of Health and Welfare (2024). Therefore, there are many chronic patients who cannot undergo rehabilitation after the acute stage. Studies have shown that home-based rehabilitation programs can be an effective option for certain medical conditions and are also perceived as being a more positive and convenient experience than hospital-based rehabilitation Arsenault-Lapierre et al. (2021); Doig et al. (2011). Although effective, patients who took part in a hospital-based rehabilitation program were shown to have better improvements to their mobility, pain, and depression than patients who only received home-based treatments Chen et al. (2023).

The effectiveness of the rehabilitation is largely based on the patient’s adherence to the prescribed treatment Hayden et al. (2005). Adherence is defined as the degree to which a patient’s behavior corresponds with the recommendations from a healthcare provider WHO (2003). Some home-based treatments may include visits from a doctor, but program adherence is harder to regulate than in a hospital setting. There are several factors that may contribute to a patient’s non-adherence.

One of the largest inhibitors to adherence is a patient’s perceived barriers. This is a patient’s own estimation of the level of challenge to a specific behavior or goal Glasgow (2020). This self-estimation may or may not accurately align with the objective challenge of the task. One study showed that patients who were prescribed two daily exercises had significantly higher levels of adherence than patients who were prescribed eight exercises Henry et al. (1999). Similarly, in a study regarding athletes’ adherence to a rehabilitation program after an injury, it was shown that the participants often forgot or claimed to not have time for the exercises Marshall et al. (2012). A part of physical rehabilitation is repeating the same exercises over an extended period of time in an effort to improve certain metrics such as range of motion or strength. These repetitive exercises are often described by the patients as being tedious and uninteresting Burke et al. (2009). This lack of motivation can inhibit rehabilitation efforts and result in delayed or impaired recovery. Home-based rehabilitation can be equally effective, but it necessitates strong patient motivation and regular follow-up appointments Frih et al. (2009).

Depending on the medical condition and severity of the injury, rehabilitation can be a slow and painful process. For example, patients who suffer from spinal cord injuries or strokes receive the highest annual hours of treatment Saumur et al. (2022). Additionally, some medical conditions may require months or years of rehabilitation to achieve full recovery Greenberg et al. (2018). This slow progress can make day-to-day results and feedback difficult to visualize. Lack of positive feedback is a key factor in a patient’s non-adherence to their physical therapy Sluijs et al. (1993).

Loneliness, especially with older adults, is a common issue with home-based rehabilitation Lykke and Handberg (2019). Further, after serious medical complications such as a stroke, patients have also been reported to suffer from depression as well Kotila et al. (1998). These negative feelings of isolation and depression can become an issue for the success of the rehabilitation since emotional support from friends and family is correlated to an increased adherence to a treatment program Levy et al. (2008).

Gamification refers to the process of adding game-like elements to a non-game-related activity Von Bargen et al. (2014). This is typically done for such reasons as increasing motivation or engagement. Studies have since been conducted to better understand how these gamification techniques can be utilized in a therapy setting. Janssen et al. described the similarities between game design and therapy design Janssen et al. (2017). They state that by using game principles, a medical specialist could better tailor a rehabilitation program to meet the needs of an individual. In practice, most gamification research targets the hand, knee, posture, and gait while being augmented by external devices such as robotic exoskeletons or virtual reality Tuah et al. (2021).

In this work, we propose a gamification interface, named Max Well-Being (MWB), that allows for different forms of input to be mapped to any in-game button command. This then creates the flexibility for a medical specialist to adjust a game to fit the medical condition and goals of the patient. The MWB system aims to demonstrate a wider potential for commercial games to be used in traditional rehabilitation methodologies. The future aim of this research is to use the MWB to improve three problems with rehabilitation; motivation, feedback, and isolation. However, the current goals of this study are to i) propose a method of selecting a commercial video game for physical rehabilitation, ii) verify the performance of the hardware and software components, and iii) determine the overall usability of the system by both medical specialists and potential end users.

## 2 Related works

There have been many studies that have developed games for specific medical conditions. The Fun-Knee is a smart knee brace that pairs with a fishing game application on a smartphone Qiu et al. (2017). In the game, users who have undergone a total knee replacement surgery can improve their range of motion by bending their knee to control the depth of a fishing lure to catch fish. Another study by Ozgur et al. that targets upper-arm rehabilitation created a tabletop maze where a user can physically move a robot through it with their hand to collect points and avoid enemies in a format that is reminiscent of the game Pac-Man Ozgur et al. (2019). These types of devices are effective in overcoming some of the limitations of conventional therapy, but their specific design limits them from being widely adopted.

The commercial video game industry has been rapidly growing over the years Palma-Ruiz et al. (2022). This industry already creates engaging and accessible user experiences. One report found that on average, people play video games 7.6 h per week Severin (2022). Therefore, this research emphasizes utilizing commercial games for rehabilitation. Research by Colder Carras et al. highlighted that many commercial games have shown potential as a therapy for mental health, neurological rehabilitation, psychotherapy, etc. Colder Carras et al. (2018). Other studies have also used commercial games and hardware for physical rehabilitation. However, these studies are usually limited to using games and systems that already have physical motion implemented as a game-play element such as the Microsoft Kinect, Nintendo Wii, Balance Board, or VR headsets Almasi et al. (2022); Marques-Sule et al. (2021); Anwar et al. (2022).

In any game, there is a theoretically infinite number of actions that get mapped to a finite number of buttons based on the system. The complexity of this mapping process can be referred to as control dimensionality (CD) Mustaquim and Nyström (2014). One of our previous studies explored the control dimensionality of different controller types and used that as a basis for designing a new controller for persons with upper limb deficiencies Hassan et al. (2022). Though effective, this process would require creating a special controller for each unique disability or rehabilitation program. Another study categorized commercial controllers by their mapping style Akyaman and Alppay (2021). The four categories were directional mapping (joystick, keypad), kinesic mapping (Microsoft Kinect), incomplete tangible mapping (Nintendo Joy-Con), and realistic tangible mapping (steering wheel). The modular nature of the proposed system aims to provide flexibility for mapping different user movements to in-game actions.

## 3 Methodology

### 3.1 System overview

#### 3.1.1 Hardware

**FIGURE 1:**
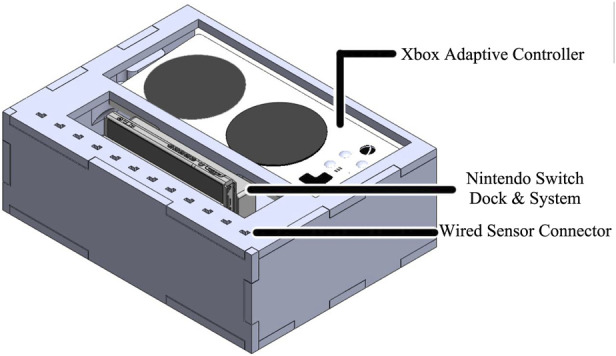
MWB system hub.

The MWB System Hub is the main enclosure that houses the main system components, Figure 1. The System Hub includes a gaming system (Nintendo Switch, Nintendo, Japan), micro-controller (Arduino Mega 2,560, Arduino, Italy), custom printed circuit board (MWB Arduino Shield), wireless transceiver (nRF24L01, Nordic Semiconductor, Norway), configurable controller (Xbox Adaptive Controller, Microsoft, United States), and USB adapter (Magic NS 1, MAYFLASH, China).

**FIGURE 2:**
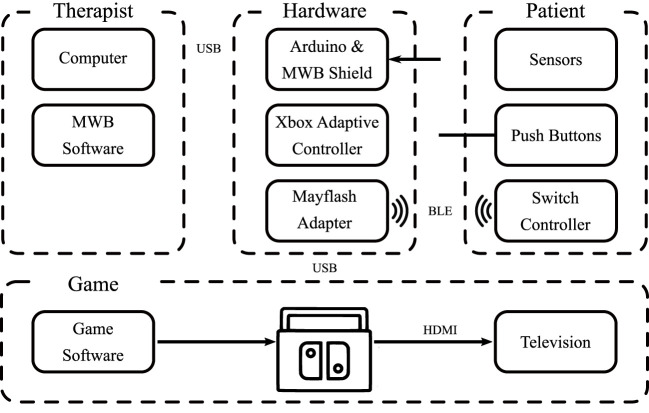
System overview.

A custom circuit was designed in the form factor of an Arduino Shield; a PCB that can be plugged directly into an existing Arduino to add additional capabilities. As a prototype, this circuit was designed with three 3.5 mm headphone jacks to allow the microcontroller to send signals directly to the Xbox Adaptive Controller. The Xbox Adaptive Controller then relayed these controls through an intermediary USB adapter that allowed for the control of the Nintendo Switch. The system overview is outlined in Figure 2.

The estimated cost of the MWB System Hub and sensors combined is $700 USD. However, the Nintendo Switch, Xbox Adaptive Controller, and 2 games alone cost $520 USD. Since the code has already been developed, the time required to implement the system would only include the time it takes to 3D print the case, connect the components, and upload the code to the device.

#### 3.1.2 Software

**FIGURE 3:**
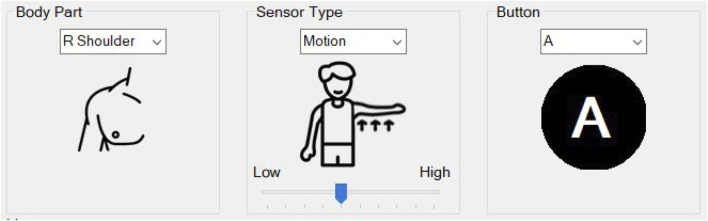
Specifying the sensor and game button settings in the program.

**FIGURE 4:**
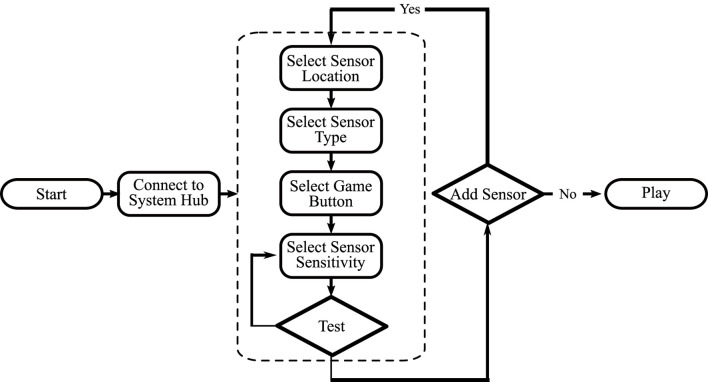
Software usage flow chart.

A program to facilitate the mapping of sensor data to in-game buttons was created using C# and Visual Studio 2022. The program was designed to be a simple user interface (UI) that would allow a medical specialist or patient to use it without extensive technical training. The software handles complex tasks and allows the user to be able to focus on the relevant features. When the program is first loaded, a welcome screen is displayed with a summary of the three steps needed to be able to use the system. The first step is a tab that connects the computer to the MWB System Hub. In code, this checks for the correct COM port and establishes a serial connection with the microcontroller. The second step takes the user to the sensor pairing screen. This screen allows for a sensor to be mapped by specifying the location of the sensor on the body, the type of sensor being used, the sensitivity of the sensor, and the in-game button to be mapped to, Figure 3. Once all specifications have been set, the user can test the sensitivity threshold by using the sensor. When the threshold has been reached, the on-screen button icon will turn green. This indicates the point in the game at which that button will be activated. The third step indicates to the user that they may close the program and focus their attention on the monitor to which the game system is connected. The user can then play the game with the control method they previously established. The software usage flow chart is shown in Figure 4.

Three key settings are needed to pair a sensor with the system; body part, sensor type, and button. The body part refers to the joint on the participant’s body where the sensor will act. If the “shoulder” is selected, the sensor can be worn around either the upper or lower arm. However, the sensor is set to detect the movement of the shoulder joint when raising or lowering the arm. The “R” and “L” notation refer to the “right” and “left” sides of the body, respectively. The “Push Button” condition is also listed here even though it is not part of the body. The sensor types refer to the three sensors that were developed for testing the system; motion, angle, and push button. The button category refers to the in-game button to which the sensors will trigger. The full list of options that are available with the current system is shown in Table 1.

**TABLE 1:** Button mapping options.

Body part	Sensor type	Button
R Shoulder	Motion	A
R Elbow	Angle	B
R Knee	Push Button	X
L Shoulder		Y
L Elbow		L
L Knee		R
Push Button		D-Pad Right
		D-Pad Left
		D-Pad Up
		D-Pad Down

Two additional features are not included in this flow chart. First, the users have a button that allows them to refer to the general usage guidelines at any point. Second, the users can change the UI to Japanese. Since the experiments took place at the University of Tsukuba Hospital in Japan, this option was selected by most participants.

#### 3.1.3 Sensors

The MWB was designed to be compatible with any standard 3 V or 5 V sensor. For the usability experiments, three different sensors were used: a motion sensor, a joint angle sensor, and an analog push button. The motion and joint angle sensors are wireless to prevent restricting the movement of the user. The push button is wired directly to the system hub.

**FIGURE 5:**
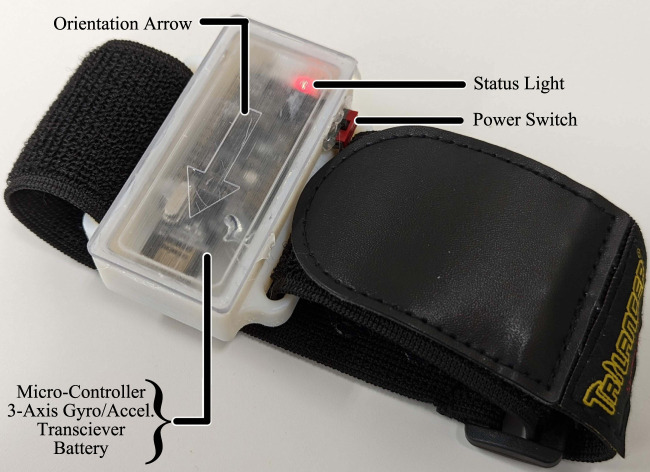
Motion sensor.

The motion sensor, shown in Figure 5, consists of a micro-controller (Adafruit Feather M0 Express - ATSAMD21 Cortex M0, Adafruit, US), 3-axis gyro accelerometer sensor (GY-521, AiPCBA Technology Co., Ltd., China), and wireless transceiver (nRF24L01, Nordic Semiconductor, Norway). These components are housed inside a 3D-printed case and can be worn by the user via a hook-and-loop strap. The sensor is powered by a 3.7v 820 mAh rechargeable Li-Ion battery. The microcontroller was programmed using the Arduino IDE. When the device is powered on, the microcontroller will continuously send data from the 3-axis sensor to the system hub. If the values exceed the threshold specified in the MWB computer software, it will trigger the desired in-game button.

**FIGURE 6:**
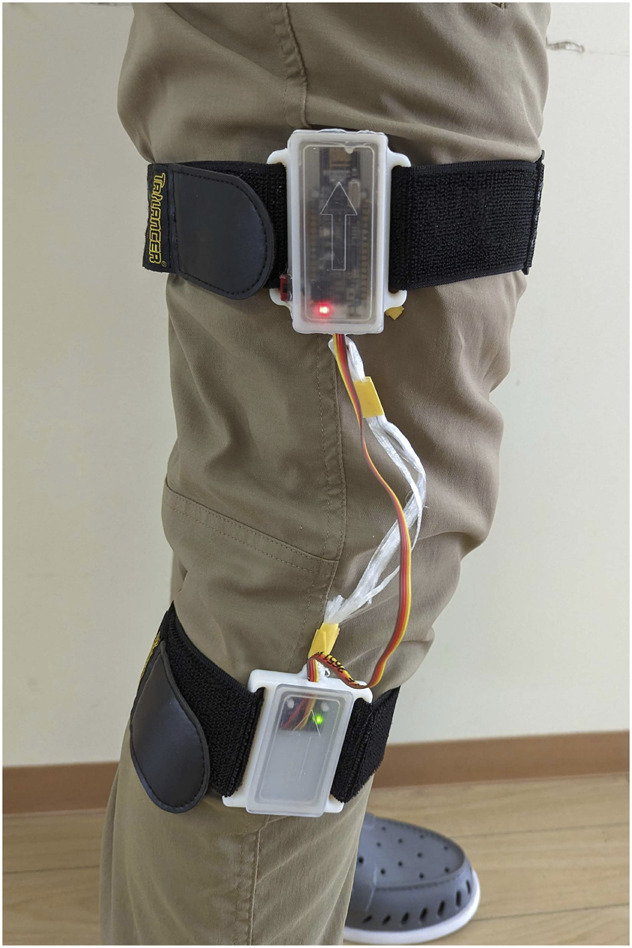
Joint angle sensor worn to measure the user’s knee angle.

The joint angle sensor uses the same components as the motion sensor with the addition of a second 3-axis gyro accelerometer. The second 3-axis sensor is mounted in a separate 3D printed case, Figure 6. This additional 3-axis sensor is physically wired to the microcontroller. There is a slightly shorter rope attaching the two cases to prevent any tension from disconnecting the cable during use. The microcontroller calculates the angle between the two sensors and transmits that data to the system hub. If the angle values exceed the threshold specified in the MWB computer software, it will trigger the desired in-game button.

A series of analog push buttons were mounted to a wooden plate. These buttons can be individually wired to the system hub. The user can press these buttons with their foot or hands to control any desired in-game button.

**FIGURE 7:**
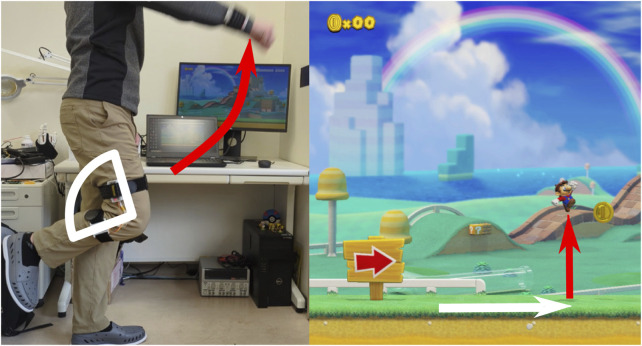
An example of using the sensors to play a Mario level.

Figure 7 shows one example of using the motion sensor and angle sensor to play a simple Mario level. When the motion sensor on the arm is raised passed a specified threshold, Mario will jump (shown in red). When the joint angle sensor on the knee exceeds the threshold, Mario will move forward (shown in white).

### 3.2 Game selection

MWB is designed to be compatible with various commercial video game software. However, this does not imply that any commercial video game would be beneficial for use in physical rehabilitation. Therefore, we propose a set of guidelines for selecting a suitable game to be used in rehabilitation. Previous studies have already proposed frameworks to evaluate games in this manner such as the Rehabilitation Gaming Model (RGM) Holmes et al. (2015). This model rates games based on player personality, game mechanics, and behavior techniques. The framework developed by Lohse et al. describes six key elements of game design that promote motivation and engagement; reward, optimal challenge, feedback, choice/interactivity, clear instructions, and socialization Lohse et al. (2013). However, these frameworks are only applicable to how engaging or entertaining a game is to a user. They do not take into consideration any practical aspects for which games would make effective rehabilitation tools. Instead, this research proposes an evaluation framework that contains the following criteria:

1. Minimal control dimensionality
2. In-game accessibility options
3. Controls suitable for slow reaction times

Bateman et al. proposed a method to calculate the CD of a game Chris Bateman (2006). One point is first given for each dimension of movement control, then each additional action (jump, attack, etc.) is 0.5 points. For example, if you consider the standard control scheme of a 2D Mario game, the CD is 3 (1 point for left-right movement, one point for up-down movement, 0.5 point for jump, and 0.5 point for fireball). The technical limitations of the MWB system currently allow for a maximum CD of 2.5. However, we propose using a slower paced game with a maximum CD of 1.5 for physical rehabilitation.

**FIGURE 8:**
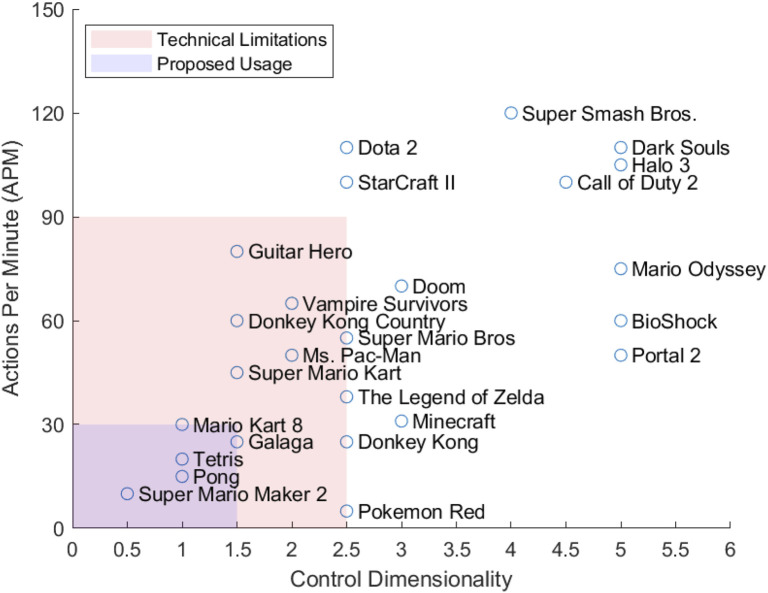
Estimated Actions Per Minute and Control Dimensionality for 25 popular video games.

Figure 8 plots 25 of the most popular games based on the relationship between the required actions per minute (APM) and the CD. APM is a metric often used in eSports as a way to measure both the cognitive and motor speeds of the players during the game. For reference, professional players in StarCraft II have been recorded to perform at an average of 267 APM while amateur players play at an average APM of 60 Krajewski (2021). As most games do not have statistics regarding the APM for a specific game, the authors estimated the APM based on game genre (e.g., fighting games are assumed to have a higher APM than a puzzle game). The CD can also be greatly affected by in game accessibility options and difficulty level. Guitar Hero is a rhythm game that can be played on a hard difficulty with up to five buttons and 300+ APM. However, on the easiest difficulty, the game uses only three buttons and can have an APM of 60–100. Since the goal of the MWB system is for rehabilitation, the authors estimated the minimum viable APM and CD for the listed games to be able to complete a level on the easiest difficulty.

Using the above guidelines, we selected two games that we believe to be suitable for the physical therapy paradigm; Super Mario Maker 2 (Nintendo Switch) and Mario Kart 8 Deluxe (Nintendo Switch). Super Mario Maker two is a game that allows the user to design their own Mario levels. This can be used to set clear goals and conditions for the patient by the medical specialist. The control dimensionality also works well. In the game’s simplest form, there is only one button, Jump (CD of 0.5). The level can be designed to move Mario automatically via in-game options. For slightly more complex exercises, Mario can be commanded to both Jump and Move Right (CD of 1). Since the level is user-made, the level can be designed to contain no enemies or obstacles. In our test levels, the level only contained coins to motivate the player to move and jump and, therefore, perform the desired exercise.

Mario Kart 8 Deluxe initially is a game with a higher control dimensionality. Due to in-game accessibility options, namely, “Smart Steering” and “Auto-Accelerate”, the game can be played with only two buttons, left and right (CD of 1). Smart Steering stops players from driving off the track and Auto-Accelerate removes the need for the player to press an extra button to move forward.

## 4 Experimental evaluations

After modifying the hardware and software interface from the design revision stage, a usability study was conducted with 10 students from the University of Tsukuba and 10 medical specialists from the University of Tsukuba Hospital. All the experimental procedures were approved by the University of Tsukuba internal review board (2021R521). Informed consent was obtained from each participant before the experiment.

Each participant was asked to complete three tasks of varying difficulty; easy, medium, and hard. The tasks were written to i) provide a clear goal, ii) have the participant use a different variety and number of sensors each time, and iii) explain the in-game buttons for participants who are not familiar with the controls. To accommodate for any learning effect, each participant was assigned the tasks in a random order. Tasks one and two used Super Mario Maker 2, while Task three used Mario Kart 8 Deluxe. The three tasks are listed below:

1. Make Mario Jump (A Button) using MOTION sensor one attached to your arm.
2. Make Mario Jump (A Button) using MOTION sensor one attached to your arm and Run (D-Pad Right) using the ANGLE sensor attached to your knee.
3. Make the car turn right using MOTION Sensor one attached to your right arm. Make the car turn left using MOTION Sensor two attached to your left arm. Use a speed boost (L Button) when the BUTTON is pressed with your foot.

During the experiment, each participant was recorded using both an external video camera and a screen recorder on the computer. These videos were manually annotated after the experiment to note any complications or errors that the participant made and the time it took to complete the task. The only intervention from the experimenter was to reset the system in between tasks and to offer simple clues or assistance if necessary. The number of times and to the extent to which assistance was provided to the participant was also recorded. The task completion time was measured from the time the participant first flipped over the instructions, touched the laptop, or picked up one of the sensors to the moment that they finished the sensor set-up and clicked on the “Play” tab.

**FIGURE 9:**
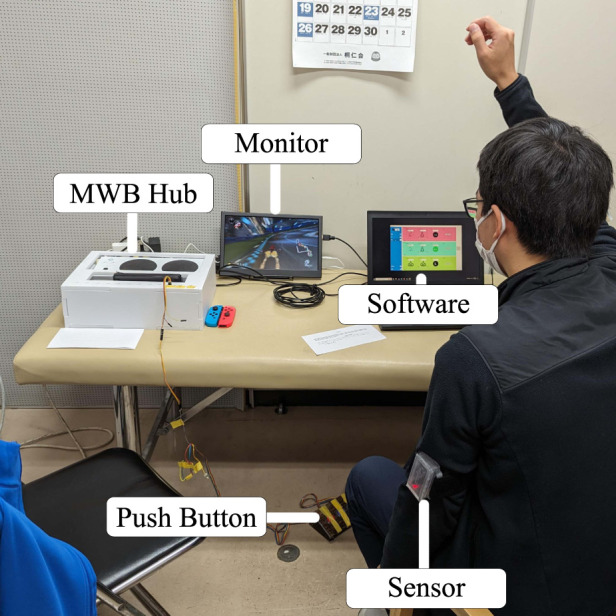
Experimental setup with a medical specialist in the University of Tsukuba Hospital.

After the participant had completed all three tasks, they were given the System Usability Scale (SUS) to rate their experience with the system. Both the tasks and the survey questions were translated into Japanese with the help of a native speaker for the convenience of the participants. The experimental setup in the University of Tsukuba Hospital can be seen in Figure 9.

### 4.1 System Usability Scale

One of the goals of the MWB system was to be easily useable by both physicians and patients to not add barriers to adherence. There are several different ways to measure the usability of a system. The System Usability Scale was chosen as the method for analyzing the usability of the system for two main reasons. First, as this survey was made freely available in 1995, there have been numerous studies that have both used the scale and analyzed it Bangor et al. (2009); Brooke (2013). This provides a good comparison for ranking our system usability to other similar products. Second, in comparison to other usability surveys, even though it is relatively short with only 10 questions, the SUS has been shown to provide the most reliable results for small sample sizes (8–12 participants) Tullis and Stetson (2004).

The survey questions in this study were adopted from the System Usability Scale and are listed in Table 2 Brooke (1995). The questions are rated on a scale of 1 (strongly disagree) to 5 (strongly agree). A SUS score is then calculated based on the question’s contribution factor and then multiplied by 2.5. This gives a final SUS score on a scale from 0 to 100.

**TABLE 2:** System usability scale.

System usability scale
I think that I would like to use this system frequently
I found the system unnecessarily complex
I thought the system was easy to use
I think that I would need the support of a technical person to be able to use this system
I found the various functions in this system were well integrated
I thought there was too much inconsistency in this system
I would imagine that most people would learn to use this system very quickly
I found the system very cumbersome to use
I felt very confident using the system
I needed to learn a lot of things before I could get going with this system

In an effort to achieve a sufficient usability level, the MWB system underwent many revisions to refine both the hardware and software prior to the experimental evaluations. These revisions were based on the feedback received from consultations with both medical care providers and average users.

## 5 Results

The first metric that was used to evaluate the usability of the system was the SUS score. The university students rated the system an average score of 69.25 ± 20.14 while the medical specialists gave an average score of 72.5 ± 17.16. No statistical difference was found between the SUS scores of the two groups.

**FIGURE 10:**
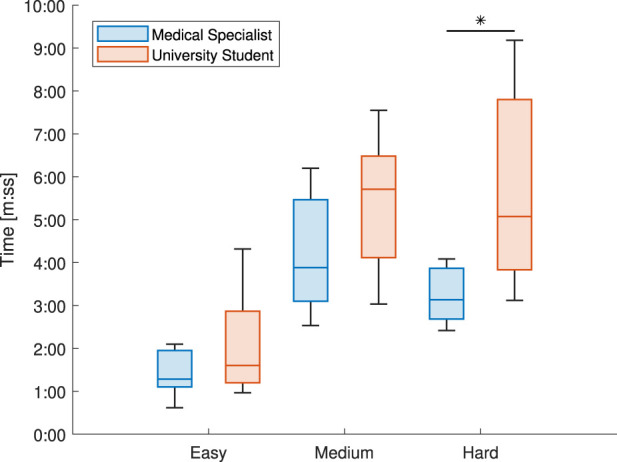
Comparing the median times needed to complete each of the three tasks between the university students and medical specialists.

The second metric to evaluate the usability was the time required by the participant to complete each of the three tasks; easy, medium, and hard. On average, the students took longer to complete the tasks than the medical specialists. These results are shown in Figure 10. Only the hard task showed a statistical difference in the task completion time between the university students and medical specialists (Wilcoxon signed-rank test: *p* = 0.0039).

All usability experiments were recorded and later annotated for analysis. In addition to any unique occurrences, some common factors were measured for all participants. The first was the number of interventions that were either requested by the participants or provided if the circumstances required it. As there was assumed to be a learning effect as the participants familiarized themselves with the system during the experiment, the number of these interventions that occurred during their first task was also documented. It was also recorded if any participant required multiple interventions per task or the experiment as a whole. The other parameter that was recorded was the number of errors. A mistake is considered an error if the participant is unable to fix it themselves or recognize it before the end of the experiment. This was most commonly forgetting to turn on the sensor or to pair the physical push button in the software. It is possible to turn on the sensors during gameplay and still have them work correctly if the pairing is still done correctly. Therefore, any error that was not able to be corrected during gameplay, i.e., forgetting to pair a sensor completely, was labeled as a critical error and denotes the failure of a task.

Out of the 30 total tasks that were completed, the student group required interventions during six of the tasks and the medical specialists required interventions during 14 of them. Out of these interventions, the number that occurred during a participant’s first task was four for the students and six for the medical specialists. One student participant required multiple interventions during the experiment, while four medical specialists required more than one intervention. Out of the total 30 tasks, 3.33% ended in a critical error for the students and 13.33% for the medical specialists. On average, the medical specialists required more interventions and made more errors than the student participants. The results are summarized in Table 3.

**TABLE 3:** Interventions and Errors.

	Student	Medical specialist
Number of Interventions (First Task)	6 (4)	14 (6)
Number of Repeat Interventions	1	4
Number of Errors (Critical Errors)	4 (1)	5 (4)
Percentage of Critical Errors	3.33%	13.33%

## 6 Discussion

### 6.1 Usability study

In regards to the SUS score, it is important to note that even though it is on a scale of 0–100, it is not a percentage. Sauro et al. found that by using a large data set of aggregated SUS scores and normalizing them, the average is 66 Sauro (2011). Another study added an 11th question to the SUS survey to attempt to correlate the SUS score with an adjective rating Bangor et al. (2009). It was found that when compared to a seven-point Likert scale, “Good” (Likert 5) was 71.4, “Excellent” (Likert 6) was 85.5, and “Best Imaginable” (Likert 7) was 90.9 on the SUS. Based on these findings, we would classify the MWB as above average and “Good” in terms of usability.

MERLIN is a telerehabilitation robotic device that was designed to support patients during post-stroke recovery at home Guillen-Climent et al. (2021). A study conducted with nine patients found high levels of satisfaction and a mean System Usability Scale (SUS) score of 71.94. ePHoRt is a web-based platform that was designed for home-based motor rehabilitation Perez Medina et al. (2019). The platform was tested with 39 participants and received a SUS score of 76.1. A study by Eguiluz-Perez et al. tested the usability of their web-based rehabilitation system with both patients and medical professionals Eguiluz-Perez and Garcia-Zapirain (2014). The patients gave a SUS score of 72 and the medical professionals gave a score of 76. The MWB system received similar scores to other comparable telerehabilitation devices.

Looking again at Figure 10 and Table 3, it is shown that the student participants took longer to complete the tasks, but had fewer interventions and errors than the medical specialist group. One of the possible reasons for this is that the medical specialists spent less time trying to figure out a problem on their own before asking for assistance. In the medical field where time is of the essence, this makes sense that more would be focused on completing the task promptly. There were also a few incidents of the medical specialists being interrupted by work-related phone calls during the experiment. The experiment could not be restarted due to the nature of the study, but there were a few interventions immediately after these calls ended to assist them in remembering their current step in the process. Eight out of the 14 task interventions occurred during the Medium difficulty task for the medical specialists, and seven of these eight interventions were due to confusion regarding the angle sensor. This is compared to the two interventions in the student group that were due to the angle sensor. It was noticed that the medical specialists operated the sensors in a much quicker motion. This could sometimes cause the sensor to not activate properly due to lag issues. The student group had more experience working with similar devices in their studies and were more lenient in their operation and troubleshooting of the device. This may again be due to differences in their respective fields, as medical specialists are more used to working with commercial-grade devices instead of prototypes.

Besides the difficulties with the angle sensor, the other most common issues during operation for both groups were forgetting to turn on a sensor, attaching the sensor upside down, or forgetting to pair the push button. The system was able to adapt and still work as intended after the sensors were turned on and corrected for orientation. Forgetting to pair the push button failed as the task was completed incorrectly. The push button may have been forgotten as it was located on the floor instead of on the table with the rest of the sensors at the start of the experiment. The placement at first was assumed to be beneficial to the user because the button would be operated by their foot during the task.

The last minor issue that occurred was confusion about the terminology in the UI and task. The authors had assumed that the “D-Pad”, or directional pad, was a familiar term for most users.

### 6.2 Limitations and safety

Since the participants of this study were university students and medical professionals in their 20 s and 30 s, there is currently a lack of evidence regarding the usability of the device by children or persons over the age of 65. Certain changes to the UI or workflow may need to be modified to better accommodate them. While the authors did their best to select two suitable games for this study, there is a need for a more thorough analysis regarding possible games that can be used for physical rehabilitation and the benefits of different game genres.

The main technical limitation of the system is its ability to interface with the gaming system. There was a need for different hardware and software components to accurately interpret and transmit the user’s actions to the corresponding in-game button commands. This process could be greatly simplified through a partnership with the gaming system manufacturer and permission to interface with the gaming system directly using Bluetooth connectivity.

One of the challenges with designing rehabilitation programs is that not all patients respond to the same treatment equally. To improve the overall adherence and effectiveness of a treatment program, research suggests that the treatment should be co-designed with the patient themselves Teo et al. (2022). The MWB is a modular system and allows for a high level of variability. Therefore, the medical specialist and patient could decide together on a game, exercise, and program that is tailored specifically for that individual’s needs. Similarly, these settings can easily be modified during the course of the physical rehabilitation program by changing the game, type of sensor, or exercise in an effort to maintain adherence and avoid any plateau effects. Additionally, since the MWB is not a robotic system, it is unable to provide any direct physical support to the user. However, there is a possibility in the future to use the MWB in conjunction with other robotic assisted rehabilitation methodologies to promote engagement and adherence.

As with any kind of the physical rehabilitation, there are inherent safety risks to be considered. The system should only be operated in an area that is free from furniture, people, and other obstructions to avoid bumping into them while moving around. A physical therapist should also be consulted to select a proper game and exercise that does not exceed the capabilities of the patient.

### 6.3 Future work

This study dealt mainly with system validation and usability. Based on the results of the SUS and video annotations, the angle sensor and UI terminology need to be improved. Once these modifications have been made, future studies plan to focus on introducing the MWB into a clinical setting and analyze the potential benefits to adherence for long-term patients.

## 7 Conclusion

This study aimed to create a modular platform to assist with the gamification of physical rehabilitation. Using the developed hardware and software system, a commercial video game can be controlled by a patient during their rehabilitation. The location and type of sensor can be specified based on the targeted exercise. We also introduced a framework with which to select commercial games that better comply with the needs of physical rehabilitation. The system received an average score of 69.25 ± 20.14 and 72.5 ± 17.16 on the System Usability Scale from university students and medical specialists, respectively. Future improvements are needed to address the issues with the angle sensor and user interface naming conventions before clinical studies begin.

## Data Availability

The raw data supporting the conclusions of this article will be made available by the authors, without undue reservation.
